# Effects of Contralateral Hip Flexion Angle on the Ober Test

**DOI:** 10.1155/2022/3349940

**Published:** 2022-12-06

**Authors:** César Hidalgo-García, Alberto Carcasona-Otal, Mar Hernández-Secorún, Hugo Abenia-Benedí, Lindsay Brandt, John Krauss, José Miguel Tricás-Moreno, Orosia Lucha-López

**Affiliations:** ^1^Unidad de Investigación en Fisioterapia, Faculty of Health Science, University of Zaragoza, Zaragoza 50009, Spain; ^2^School of Health Sciences, Oakland University, Rochester, MI, USA

## Abstract

The Ober test is an orthopedic evaluation procedure used to assess for tightness of the tensor fascia latae (TFL) and iliotibial band (ITB). Multiple versions of this test have been described using different degrees of contralateral hip joint flexion to stabilize the pelvis. The aim of this study was to analyze the hip range of motion (ROM) in the frontal plane and perceived tension produced during the Ober test using four different angles of contralateral hip flexion prepositioning. The secondary objective was to analyze the differences in the Ober test with different contralateral hip flexion angles according to limb dominance. This cross-sectional study included healthy individuals aged 18 years or older. The Ober test was performed on the right and left leg of each participant with the contralateral hip joint stabilized at 0° flexion, 45° flexion, 90° flexion, and maximal flexion. Hip range of motion in the frontal plane (abduction or adduction) was measured using a digital inclinometer. Three measurements were performed on each limb for every angle of contralateral prepositioning, using the average of the three measurements for statistical analysis. Participants were asked to report the location of any perceived tension and the intensity of tension using a Numeric Rating Scale during the test. Twenty-eight participants (17 men and 11 women) were examined. Significant differences in the Ober test hip ROM in the frontal plane (*p* < 0.01) were observed when comparing different angles of contralateral hip flexion prepositioning. Significant differences between tests were also present for intensity of perceived tension (*p* ≤ 0.001), except for the intensity of perceived tension between 0° and 45°. No statistically significant differences were observed related to limb dominance (*p* > 0.05) or gender (*p* > 0.05), except for the Ober test at 0° (*p* < 0.001) which was higher in men (9.61° ± 5.01°) than in women (5.05° ± 2.87°). Greater contralateral hip flexion prepositioning during the Ober test results in decreased hip adduction ROM in the tested limb and greater perceived tension in the region of tensor fascia latae-iliotibial band.

## 1. Introduction

The dysfunction of the mobility of the hip abductor muscles have been associated to lumbopelvic [1–4], hip [4, 5], and knee [6–8] dysfunctions. The Ober test is a commonly clinical procedure designed to measure the length of the iliotibial band and the hip abductor muscles inserted into it, mainly gluteus maximus and tensor fascia latae [9].

The Ober test is performed with the patient in side lying and measures the hip ROM after the examiner moves the tested limb from an abducted and 0° extended position into adduction of the superolateral hip. The literature indicates that the knee of the tested side can be flexed 90° (Ober's test) or extended (modified Ober's test), with more restriction of the test mobility during the Ober Test version [9]. The test is completed and the ROM measured when either the adduction ROM reaches the end of the movement and/or the pelvis starts to tilt downward. Kendall et al. judged the Ober test as positive if the thigh did not reach a horizontal level [10]. However, the literature has reflected differences in the normative values of the Ober test, even showing a critical criterion for the Ober test of 23.2° of adduction [11].

While participant's characteristics could explain some of the differences reported in the literature, additional explanations relating to pelvic stabilization when performing the test could also explain these ROM differences. Stabilization of the pelvis may be performed manually by the examiner and via the flexion positioning of the contralateral hip joint. Thus, 0° [11], 45° [9, 12], and 90° [13, 14] of contralateral hip flexion have reported in the literature. Hamberg et al. validated, using radiographs, the use of maximum contralateral flexion of the hip to reduce unwanted anterior pelvic tilting during stretching of rectus femoris [15]. No study has evaluated the influence of contralateral hip flexion including maximal hip flexion on the ROM and symptom provocation during the Ober test.

This study's objective was to analyze the effects of contralateral hip flexion on the hip range of motion (ROM) in the frontal plane and patient perceptions of tension during the Ober test. Our hypothesis was that greater contralateral hip flexion during the Ober test would result in less hip adduction ROM and a greater sense of stretching during the test. The secondary objective was to analyze differences in the Ober test with varying contralateral hip flexion angles according to limb dominance.

## 2. Materials and Methods

### 2.1. Ethics

The study was conducted according to the Declaration of Helsinki and approved by the local ethics committee from a local university (06/2022; PI22/103). Informed consent for publication was provided for all figures within the manuscript.

### 2.2. Design

This study used a cross-sectional study design and was carried out with a convenience sample recruited through an advertisement at a university. Samples were collected from volunteers on a first-come, first-serve basis. Data was collected from November 2021 to March 2022 at University of Zaragoza (Zaragoza, Spain). Participants were excluded if they were less than 18 years old, had persistent pain (more than seven days in the last three months) in the lumbar spine or lower extremity, or a diagnosis of lumbar, hip, or knee pathology. Participants were advised not to perform physical activity 4 hours before the assessment. All participants signed an informed consent form prior to the evaluation.

### 2.3. Procedures

Examination was carried out at the University of Zaragoza. All measurements were carried out in the same room of the Faculty of Health Sciences (University of Zaragoza) at a room temperature of 20-22° Celsius. Sociodemographic data were collected first and then ROM measurements were taken following a warm-up of three-minutes walking and three lumbo-pelvic and hip movements in sitting and standing position. Participants were asked to perform three active hip abduction and adduction movements until the end-range. All participants were evaluated and measured by the same physical therapist, who had three years of clinical experience. Preliminary work was carried out to standardize the measurement protocol, consisting in four sessions for the standardization of the measurement protocol.

**Figure 1:**
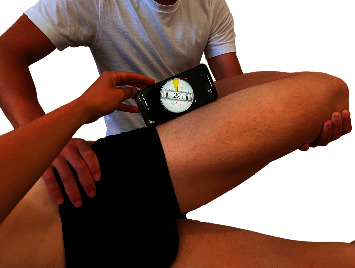
Inclinometer measurement.

**Figure 2:**
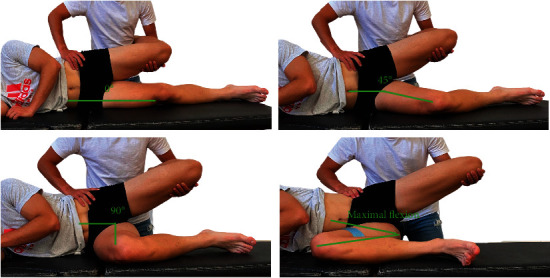
Ober's test with different contralateral hip flexion (0°, 45°, 90°, and maximal contralateral hip flexion).

The Ober test was performed with the patient in side-lying and the examiner positioned behind the patient's pelvis. The participant's superolateral pelvis was stabilized by one therapist's hand while therapist's other arm cradled the leg and the knee of the patient in 90° of flexion. The participant's superolateral thigh was brought into hip abduction and 0° of extension and then moved into hip adduction in the frontal plane. The movement was stopped when an end-feel was reached by the evaluator or when the superolateral pelvis started to move caudally. This technique has been shown to be reliable (ICC = 0.82-0.94) [9, 12]. A mobile phone with a clinometer (LIS302DL accelerometer) application was positioned in the lateral part of the thigh (in the midpoint between the anterior superior iliac spine and the lateral condyle of the femur) to measure the ROM (Figure 1). The Ober test was repeated three times in each of the contralateral hip positions, 0°, 45°, 90°, and maximal flexion stabilized with a belt (Figure 2). The final ROM was the average of the three repetitions. After completing the third repetition, the participant was asked to rate their tightness using a 0-10 scale (tightness NRS) as well as the location of the tightness in both lower extremities. The contralateral hip flexion positions for each Ober's test were randomly performed using the “Alazar” web app (http://Alazar.info, Spain).

### 2.4. Variables

The dependent variables were hip ROM in the frontal plane (“+” for abduction ROM and “-” for adduction ROM) during the Ober test, tightness symptoms measured using a Numeric Rating Scale (NRS) (“0” for no tension to “10” for maximal tightness), and location of the tightness (anterior, posterior, lateral, or medial side of the thigh, another area, or no tension). Independent variables were sex, gender, weight, height, physical activity (IPAQ short form), limb dominance, and the degrees of contralateral hip flexion.

### 2.5. Statistical Analysis

All analyses were carried out using IBM SPSS statistics 25.0 (IBM, Armonk, NY, USA). The Shapiro-Wilk test was used to evaluate the normality of variables with a significance level of *p* < 0.05 was established. For the descriptive study of quantitative variables (ROM and tightness NRS), the mean and standard deviation for the parametrical variables and the median and maximum/minimum for the nonparametrical variables were calculated. For the descriptive study of qualitative variables (sex, limb dominance, and location of the tightness) absolute frequencies and percentages were calculated. A one-factor ANOVA was performed to compare ROM and tightness NRS based on the four contralateral hip flexion positions. Sex and limb dominance differences in ROM and tightness NRS were analysed using the Student *t*-test for parametric variables and the Mann–Whitney *U*-test for nonparametrical variables. Differences in the location of the tightness according to the different contralateral hip flexion positions during the Ober test were evaluated with Pearson's chi-squared test.

## 3. Results and Discussion

Twenty-eight participants (17 males and 11 females) with a median age of 21 years old (19/41) met the criteria to participate in the study. The median weight and height of the subjects were 71 kgs (55/86) and 173 cms (158/187), respectively. 39.3% of the subjects had a high level of physical activity, 25% moderate level, and 35.7% low level.

No differences were found between limb dominance (100% right). Fifty-six lower limbs were analyzed. No significant differences were present between male and females (*p* > 0.05) except for abduction ROM in the 0° Ober test position (*p* < 0.001) which was higher in men (9.61° ± 5.01°) than in women (5.05° ± 2.87°).

### 3.1. Ober's Test ROM and Tightness NRS with 0°, 45°, 90°, and Maximal Contralateral Hip Flexion

ROM values and tightness feelings are presented in Table 1. The most limited ROM was 19.94° ± 4.94° of hip abduction with maximal contralateral hip flexion and the greatest ROM was 7.81° ± 4.82° of hip abduction in 0° of contralateral hip flexion. With 0° of contralateral hip flexion, Ferber et al. showed more mobility into hip adduction (23.2°) [11]. With 45° of contralateral hip flexion, Wang et al. [16] and Reese and Bandy [12] showed 17.8° and 18.9°, respectively, in young and asymptomatic samples. With 90° of contralateral hip flexion, Herrington et al. [13],, using pressure biofeedback to detect the onset of pelvic motion, showed an adduction ROM of 9.91°. The values of Gadjosik et al. [9] 6° abduction in women and 4° in men with a 90° contralateral hip flexion angle are slightly lower than the 14.8° abduction of our sample. Variations in the methods used to control pelvic movement and identify the end of hip adduction are likely sources of variability in the Ober test ROM within the literature.

This study did not find statistically significant ROM differences between male and females, except at 0° (*p* < 0.001) which was higher in men (9.61° ± 5.01°) than in women (5.05° ± 2.87°). Gadjosik et al. suggested that one potential explanation could be the larger weight of the lower extremity of the male compared to the female [9]. To minimize this phenomenon, the examiner held the weight of the lower extremity and passively mobilized into adduction during this study. This change in methodology may also be responsible for variations in the final value of the test among the studies. The minimal tightness during the Ober test was with 0° of contralateral hip flexion (0.36 ± 0.78) and the greatest tightness was with maximal contralateral hip flexion (2.73 ± 1.58). The reduction of ROM, increasing tightness, and the provocation of tightness location in the area of the lateral side of the hip and thigh indirectly suggest that maximal contralateral hip flexion provides more stabilization to the pelvis compared to other contralateral hip flexion angles.

Table 2 shows the statistical analysis of the ROM and tightness feeling during the Ober test at different contralateral hip flexion angles. Increasing lumbopelvic stability via increasing contralateral hip flexion demonstrated a statistically significant reduction in ROM during the Ober test in a young and asymptomatic sample. This is similar to Hamberg et al. who showed the same results with maximal hip flexion during rectus femoris length test [15]. As indicated previously, the literature reflects this finding in the Ober test ROM, with values ranging from more than 20° of adduction with 0° of contralateral hip flexion and 6° abduction with 90° of contralateral hip flexion. Our study showed for the first time the restriction of the Ober test ROM values with increasing contralateral hip flexion values in the same sample, including maximal contralateral hip flexion.

Table 2 also shows a statistically significant increase tightness reported by participants with increasing contralateral hip flexion during the Ober test except for the comparison from 0° to 45°. Participants reporting tightness symptoms in the lateral thigh moved from 6 (11%) for 0° of contralateral hip flexion to 31 (56%) participants for maximal contralateral hip flexion (*x*^2^Pearson = 37.59; *p* < 0.05). Similarly, the number of participants reporting no tightness during the Ober test was reduced from 21 (38%) to 10 (17%) for maximal contralateral hip flexion. The number of participants reporting tightness in the anterior thigh reduced from 29 (51%), 31 (56%), and 27 (49%) in 0°, 45°, and 90°, respectively, to 15 (27%) in maximal contralateral hip flexion. The Ober test is considered to be an indirect measure of the length of the iliotibial band [13]. In fact, Wang et al. identified morphological changes of the iliotibial band during the Ober test using ultrasonography [16]. In our study, greater stretching in the lateral side of the thigh was reported during maximal contralateral hip flexion. More studies are needed to explore the effects of Ober test with maximal contralateral hip flexion as a stretching technique for therapeutic purposes.

Several limitations are present within this study. The asymptomatic or subclinical condition of our sample and the lack of a-priori sample power analysis limit the generalization of the results to the population and patients with lumbopelvic, hip, and knee pathologies. More studies are needed to examine the effect of maximal contralateral hip flexion during the Ober test in these clinical subgroups and with the application of the Ober test with an extended knee [9]. Evaluator bias could also be present during the measurements. A training consisting in 4 (four) sessions for the group of evaluators was developed and especially focused on the standardization of the stabilization of the pelvis. Due to the heterogeneous methodology used when the performing of the Ober test represented within the literature, it is difficult to compare the results of this study with other studies.

### 3.2. Future Studies

More studies are needed to standardize the Ober test and establish the psychometric properties of the Ober test with maximal contralateral hip flexion. The values of the Ober test in patients with patellofemoral pain syndrome have been reported to be reduced compared to controls [17]. However, no difference in the Ober test ROM with 45° contralateral hip flexion was found in a sample with iliotibial band syndrome [18]. More studies are needed to establish the relationship of gender and age in asymptomatic and symptomatic populations with maximal contralateral hip flexion during the Ober test.

## 4. Conclusions

Increasing contralateral hip flexion during Ober's test reduced the Ober test ROM and increased and focused the tightness feeling in the lateral side of the thigh in a healthy and young sample.

## Figures and Tables

**Table 1:** Descriptive values of ROM, tightness feeling, and location of tightness during the Ober test with different contralateral hip flexion.

Contralateral hip flexion (*n* = 56)	Variable	Value	Location of tightness (fi: %)	
0°	ROM	7.81° ± 4.82°	LST	6 (11%)
	Tightness NRS	0.36 ± 0.78	AT	29 (51%)
			No tension	21 (38%)

45°	ROM	10.86° ± 4.20°	LST	7 (13%)
	Tightness NRS	0.57 ± 0.78	AT	31 (55%)
			No tension	18 (32%)

90°	ROM	14.84° ± 4.31°	LST	16 (29%)
	Tightness NRS	1.45 ± 1.43	AT	27 (49%)
			No tension	13 (24%)

Maximal flexion	ROM	19.94° ± 4.94°	LST	31 (56%)
	Tightness NRS	2.73 ± 1.58	AT	15 (27%)
			No tension	10 (17%)

ROM: range of movement (°); NRS: Numeric Rating Scale (0-10); Fi: absolute frequency; %: percentage; LST: Lateral Side of Thigh; AT: Anterior Thigh.

**Table 2:** Comparative values of ROM and tightness feeling during the Ober test at different contralateral hip flexion angles.

Ober test changes	ROM (*n* = 56)		Tightness NRS (*n* = 56)	
	Average (CI 95%)	*p*	Average (CI 95%)	*p*
0°-45°	-3.05° (−5.3°/−0.8°)	0,003	-0.2 (−0.8/−0.4)	0,779
0°-90°	-7.0° (−9.3°/−4.8°)	0,000	-1.1 (−1.7/−0.5)	0,000
0°-max flex	-12.1° (−14.3°/−9.9°)	0,000	-2.4 (−3.0/−1.8)	0,000
45°-90°	-4.0° (−6.2°/−1.7°)	0,000	-0.9 (−1.5/−0.3)	0,001
45°-max flex	-9.1° (−11.3°/−6.9°)	0,000	-2.2 (−2.7/−1.6)	0,000
90°-max flex	-5.1° (−7.3°/−2.9°)	0,000	-1.3 (−1.9/−0.7)	0,000

ROM: range of movement (°); NRS: Numeric Rating Scale (0-10); Flex: contralateral hip flexion.

## Data Availability

The data of the study are available from the corresponding author.
